# Ultrasound-assisted extraction optimization of polyphenols from *Boletus bicolor* and evaluation of its antioxidant activity

**DOI:** 10.3389/fnut.2023.1135712

**Published:** 2023-03-29

**Authors:** Dong-Bao Hu, Rui Xue, Xiao-Cui Zhuang, Xin-Sha Zhang, Sheng-Li Shi

**Affiliations:** School of Chemistry, Biology and Environment, Yuxi Normal University, Yuxi, China

**Keywords:** ultrasound-assisted extraction, *Boletus bicolor*, polyphenols, UPLC-MS analysis, DFT, antioxidant activity

## Abstract

**Introduction:**

*Boletus bicolor (B. bicolor)* mushrooms are widely consumed as a valuable medicinal and dietary ingredient in China, but the active ingredients of this mushroom and their extraction methods were not extensively studied.

**Methods:**

In this paper, we propose an optimized ultrasound-assisted extraction (UAE) method to detect natural antioxidant substances in *B. bicolor*. The antioxidants were quantitatively and quantitatively determined using UPLC-MS, the polyphenols were evaluated based on response surface methodology (RSM), and density functional theory (DFT) calculations were performed.

**Results:**

The results showed that the optimal extraction was obtained under the following conditions: ethanol concentration 42%; solvent to solid ratio 34:1 mL/g; ultrasonic time 41 min; and temperature 40°C. The optimized experimental polyphenol value obtained under these conditions was (13.69 ± 0.13) mg/g, consistent with the predicted value of 13.72 mg/g. Eight phenolic compounds in the extract were identiffed by UPLC-MS: syringic acid, chlorogenic acid, gallic acid, rosmarinic acid, protocatechuic acid, catechin, caffeic acid, and quercetin. Chlorogenic acid exhibits the highest HOMO energy (−0.02744 eV) and the lowest energy difference (−0.23450 eV) among the studied compounds, suggesting that the compound might be the strongest antioxidant molecule. Eight phenolic compounds from the *B. bicolor* signiffcantly inhibited intracellular reactive oxygen species (ROS) generation, reduced oxidative stress damage in H_2_O_2_-induced HepG-2 cells.

**Discussion:**

Therefore, it was confirmed that the UAE technique is an efficient, rapid, and simple approach for extracting polyphenols with antioxidant activity from *B. bicolor*.

## 1. Introduction

The human organism produces many kinds of free radicals during life, and they have strong oxidative capacity. If these active free radicals are not removed in time, various enzymes in the body may be impaired, causing metabolic disorders and leading to diseases (1–3). Reactive oxygen species (ROS) are among the most vital endogenous free radicals, produced by oxidative respiration in the normal cellular metabolism (4). If ROS levels exceed the body’s internal antioxidant threshold and are not cleared quickly, and they attack body tissues, organs and cells, causing chronic diseases such as in inflammation, aging, and cancer (5). The phenolic compounds are an important group of secondary plant metabolites which can scavenge free radicals in the body, including phenolic acids, flavonoids, distyrenes, and lignans. They are recognized as natural antioxidants for preventing various diseases, such as cardiovascular, anti-inflammatory (6). Phenolic compounds recognized as natural antioxidants in mushrooms can maintain normal metabolic activities by regulating the level of free radicals and reducing the damage in the organism occurring at molecular and cellular levels (6–8). These compounds are mainly attributed to phenolic acids, lignans, stilbenes, and oxidized polyphenols (9). As a good source of polyphenols, mushroom extracts have been found to complement industrialized products to replace synthetic antioxidants (10). For centuries, they were consumed for food or medicine as part of the human diet (11–13). Over the past 2 decades, the health-promoting effect of mushrooms has attracted increasing attention due to the wide range of secondary metabolites in fruiting bodies.

*Boletus bicolor* is a basidiomycete mushroom species widely used as delicacy food, and its extract is traditionally used to treat several human ailments (14, 15). As a kind of delicious wild mushroom, *B. bicolor* contains various beneficial components, including steroids, terpenoids, alkaloids, phenols, and proteins, and has many beneficial properties, such as enhancing human immunity, anti-tumor, antioxidant, and antibacterial (16, 17). To the best of our knowledge, the antioxidant activities obtained from wild edible fungal *B. bicolor* extracts were rarely explored, so effective extraction of polyphenols from *B. bicolor* should be helpful for its utilization. Common polyphenol extraction methods include soxhlet extraction, room temperature extraction, ultrasound-assisted extraction, microwave-assisted extraction, and enzymatic-assisted extraction (17). Conventional extraction procedures, such as percolation, maceration, and reflux, often utilize organic solvents and need a significant volume and a long extraction period. Previous studies demonstrated that conventional methods for extracting bioactive compounds are time-consuming, resulting in low yields with low amounts of bioactive compounds. Novel and environmentally friendly extraction technologies have been suggested to overcome these problems. Ultrasound-assisted extraction (UAE) is appropriate for the extraction of thermolabile and unstable compounds and for the extraction of various natural plant metabolites. The UAE has attracted significant research interest due to many advantages over conventional methods, including shorter extraction time, lower volume of organic solvents, and higher extraction yields of target components (18, 19).

The present study aims to shed more light on the UAE extraction of poorly understood *B. bicolor* mushrooms using response surface methodology (RSM). The main phenolic compounds in the mushroom were identified by UPLC-MS. Then, the mechanism of antioxidant activities was explained based on the results of the DFT computational method, cytoprotective activities assay of eight main phenolic compounds extracted from *B. bicolor* were used for validation of DFT calculations. Next, the antioxidant activities were evaluated by five methods, including DPPH scavenging activity, ABTS scavenging activity, OH scavenging activity, NO_2_^−^ scavenging activity, and ferric-reducing antioxidant power.

## 2. Materials and methods

### 2.1. Mushroom material

*Boletus bicolor* was collected from Yuxi City, Yunnan Province, China. The mushroom material was dried under ventilation until reaching a constant weight. The sample was powdered and stored in an air-tight container for further use.

### 2.2. Chemicals

2, 2-diphenyl-1-picrylhydrazyl (DPPH), ABTS [2, 2′–azinobis (3-ethylbenothiazoline-6-sulphonic acid)], and polyphenolic standards (protocatechuic acid; catechin; syringic acid; rosmarinic acid; gallic acid; chlorogenic acid; caffeic acid; and quercetin) were purchased from Sigma–Aldrich. Analytical grade ethanol, Folin–Ciocalteu reagent, and methanol were purchased from J. T. Baker (Phillipsburg, United States). Other analytical grade chemicals were supplied from Xilong Chemical Factory (Sichuan, China).

### 2.3. Extraction of polyphenols

#### 2.3.1. Single-factor experiments

To evaluate the effect of each factor on the antioxidant activity of the extract of *B. bicolor*, we investigated different ethanol amounts (10, 20, 30, 40, 50, 60, 70, and 80%), liquid-to-solid ratios (10,1, 15:1, 20:1, 25:1, 30:1, 35:1, 40:1, and 45:1), ultrasonic times (20, 25, 30, 35, 40, and 45 min), and ultrasonic temperatures (30, 35, 40, 45, 50, and 55°C) as single-factor variables in the experimental design (20).

#### 2.3.2. Response surface methodology experiments

Based on single-factor experiments, a four factor-three-level response surface analysis method was used to optimize the extraction process of polyphenols from *B. bicolor*. The RSM was applied with a Box–Behnken Design (BBD), and the optimized conditions are shown in Table 1.

**Table 1:** Factors and levels of response surface analysis.

Factors	Levels		
	−1	0	1
X1: Volume fraction of ethanol (%)	30	40	50
X2: Liquid–solid ratio (mL/g)	30	35	40
X3: Ultrasonic time(min)	35	40	45
X4: Ultrasonic temperature(°C)	35	40	45

#### 2.3.3. Soxhlet extraction

3.0000 g of *B. bicolor* powder sample was weighed accurately and put into a sorption extractor wrapped with qualitative filter paper. The extraction was carried out with 120 mL of 40% ethanol at 95°C for 4 h to obtain polyphenols.

#### 2.3.4. Maceration extraction

3.0000 g of *B. bicolor* powder sample was mixed with a 40% ethanol solution (200 mL). The extraction was conducted in a dark place, sealed with plastic wrap, and reacted for 24 h. The mixture was centrifuged for 20 min to obtain polyphenols.

### 2.4. Identification of phenolic compounds by UPLC-MS

Phenolic compounds in the extract were characterized by UPLC-MS analysis. Syringic acid, chlorogenic acid, gallic acid, rosmarinic acid, protocatechuic acid, catechin, caffeic acid, and quercetin were used as standards. The standard solutions of the phenolic compounds were prepared by dissolving them in methanol at concentrations of 0.505, 0.510, 0.515, 0.520, 0.515, 0.520, 0.525, and 0.530 mg/mL, respectively. The standard curves were plotted with the concentration as the horizontal coordinate and the peak area as the vertical coordinate, showing good linearity for all eight phenolic compounds (*r* > 0.99) (21). The polyphenolic extracts were analyzed and determined using a 4500 QTRAP triple quadrupole liquid chromatograph (AB SCIEX).

### 2.5. DFT study of the antioxidant mechanism of phenolic compounds

Gaussian 09 software was used to calculate the energies of HOMO and LUMO orbitals for the eight phenolic compounds of the *B. bicolor* mushroom. The eight phenolic compounds were calculated by the DFT at the M062X/6–311++G** level. Firstly, optimization was carried out; then, the frequencies were also implemented at the same level, which has no imaginary frequencies. Finally, the HOMO and LUMO orbitals were depicted, and the orbital energies were calculated.

### 2.6. Phenol compounds on H_2_O_2_-induced ROS concentration in HepG2 cells

Human hepatocellular carcinoma (HepG2) cells were obtained from Kunming Cell Bank and cultured in DMEM complete culture medium (supplemented with 10% FBS and 1% penicillin/streptomycin) at 37°C in 5% CO_2_. Further assays were processed once the cells reached 80–90% confluence. The cytotoxicity of eight polyphenolic compounds (1.88, 3.75, 7.5, 15, and 30 μM) was assessed (22). First, HepG2 cells were inoculated into 96-well culture plates at 5 × 10^3^ cells/well and cultured for 24 h. Second, the cell medium was changed with eight polyphenolic compounds (0, 1.88, 3.75, 7.5, 15, and 30 μM) and cultivated for 20 h. Next, remove the cell culture medium and add 200 μL of MTT solution (0.5 mg/mL, dissolved in PBS) and incubate for 4 h. Then, 150 μL of dimethyl sulfoxide (DMSO) was added and absorbance was measured at 490 nm using a micrometer plate reader. Cell viability results demonstrated that the eight polyphenolic compounds were not toxic to HepG2 cells at all tested concentrations of 1.88, 3.75, 7.5, 15, and 30 μM.

Reactive oxygen species (ROS) production in H_2_O_2_-induced HepG2 cells was assessed with reference to previous studies (23). First, cells (2.5 × 10^5^ cells/well) were placed in 2.0 ml of six-well plates for 24 h. Then, 2.0 mL of phenolic compounds (1–8, 0, and 30 μM) were replaced with cell culture medium and incubated once again for 24 h. After treating HepG2 cells with H_2_O_2_ (1.0 mM) for 8 h, the cell culture medium was removed and the cells were obtained. After that, cells were washed three times with chilled PBS and 1.0 mL DCFH-DA (10 μM, dissolved in pre-chilled PBS) was added and incubated for 20 min at 37°C in dark conditions. Upon incubation, cells were assessed by flow cytometry (Guava® easyCyte 6–2 L, Millipore, Billerica, United States).

### 2.7. Determination of antioxidant capacity

#### 2.7.1. DPPH radical scavenging assay

The scavenging effect of the polyphenol extract was slightly modified according to the previously described procedure (24). The polyphenol extract was prepared at different mass concentrations. A 100 μmol/L DPPH working solution was obtained by accurately weighing 0.0050 g of DPPH with absolute ethanol. Then, 3 mL of the DPPH working solution and 1 mL of the sample extract at different concentrations were added to a 10 mL colorimetric tube and reacted in the dark for 30 min. The absorbance value, *A*_0_, was measured at a wavelength of 540 nm. The *A*_1_ absorbance value was measured by reacting with 3 mL of DPPH instead of the sample solvent. The scavenging capacity was compared with that of ascorbic acid. The DPPH radical scavenging activity was calculated according to the following formula:


$$\%\mathrm{Inhibition of DPPH radical activity}=\left[\left(A_{0}-A_{1}\right)/A_{0}\right]\times 100\%$$

Where *A*_0_ is the absorbance of the DPPH, and *A*_1_ is the absorbance of the sample and the positive control.

#### 2.7.2. ABTS radical scavenging assay

The ABTS radical scavenging capacity assay was performed using the previous method with slight modification (24). A 7 mmol/L ABTS solution and a 4.9 mmol/L potassium persulfate solution were prepared. Then, the two solutions were mixed and placed in the dark for the reaction for 14 h. The ABTS working solution was diluted at 734 nm. 3 mL of the ABTS working solution and 0.2 mL of the sample solution were added to a 10 mL colorimetric tube, and the absorbance *A*_1_ was measured at 734 nm after 6 min in the dark. Next, using the sample solvent instead of the sample and 3 mL of the ABTS working solution, the absorbance value *A*_0_ was determined. The ABTS radical scavenging activity was calculated according to the following formula:


$$\%\mathrm{Inhibition of ABTS radical activity}=\left[\left(A_{0}-A_{1}\right)/A_{0}\right]\times 100\%$$

Where *A*_0_ is the absorbance of ABTS, and *A*_1_ is the absorbance of the sample and the positive control.

#### 2.7.3. OH radical scavenging assay

According to the reported method (24), 1 mL of a sample solution at different concentrations, 1 mL of a 9 mmol/L ferrous sulfate solution, and 1 mL of a 9 mmol/L salicylic acid-ethanol solution were mixed. Next, 1 mL of an 8.8 mmol/L H_2_O_2_ solution was added, and the reaction was thoroughly mixed for 30 min. Then, the absorbance value (*A_s_*) was measured at 510 nm. The absorbance *A*_0_ was measured with the sample solvent instead of the sample solution, the absorbance *A*_x_ was determined with the sample solvent instead of the H_2_O_2_ solution. The clearances were calculated according to the following equation.


$$\mathrm{OH}\;\mathrm{Radical Scavenging Assay}\%=\left[1-\left(A_{s}-A_{x}\right)/A_{0}\right]\times 100\%$$

#### 2.7.4. NO_2_^−^ scavenging assay

According to the previous method (24), 2 mL of a 5 mg/L NaNO_2_ solution and 2 mL of sample solutions at different concentrations were added to a 25 mL colorimetric tube. Then, 1 mL of a 4 g/L p-aminobenesulfonic acid solution was added to a water bath at 25°C and kept for 30 min. After thoroughly mixing, the solution was placed in the water bath at 25°C for 5 min. Next, 1 mL of a 2 g/L naphthalene ethylenediamine hydrochloride solution was added, and the volume was fixed to the scale with distilled water. After fully shaking, the absorbance was measured after 15 min in the water bath at 25°C. The absorbance *A_j_* was measured at a wavelength of 538 nm. *A_0_* is the control absorbance and *A_i_* is the sample absorbance. The clearance rate was calculated according to the following formula:


$${\mathrm{NO}}_{2}{}^{-}\;\mathrm{Scavenging Assay}\%=\left[1-\left(A_{j}-A_{i}\right)/A_{0}\right]\times 100\%$$

#### 2.7.5. Ferric reducing power assay

According to the previous method (25), 2 mL of the sample solution at different concentrations, 2 mL of a phosphate buffer solution (pH = 6.6), and 2 mL of a 1% potassium ferricyanide solution were mixed and reacted for 20 min. After cooling with flowing water, 2 mL of a 10% trichloroacetic acid solution was added. Then, 2 mL of the above solution was taken and reacted with 0.2 mL of a 0.1% ferric chloride solution in the dark for 10 min. Next, the absorbance of the mixture was measured at a wavelength of 700 nm in three parallel experiments.

### 2.8. Statistical analysis

All the experiments were performed in triplicate, and the results are shown as mean ± SD. The statistical analysis of the model was performed with SPSS 22.0 and plotted with Origin 2018 software. The response surface map was obtained using Design-Expert 11.1.1. In the antioxidant experiments, IC_50_ represents the sample concentration when the scavenging rate reaches 50%, and EC_50_ represents the sample concentration when the absorbance value is 0.5 in the relative reducing power.

## 3. Results and discussion

### 3.1. Single-factor experiments

#### 3.1.1. Effect of the ethanol concentration on TPY

**Figure 1:**
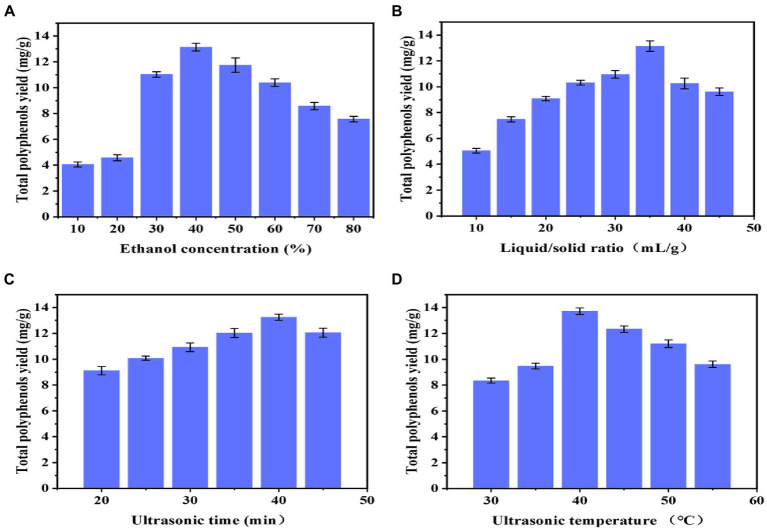
Single-factor experiments results: **(A)** Ethanol concentration; **(B)** Liquid/solid ratio; **(C)** Ultrasonication extraction time; and **(D)** Ultrasonication extraction temperature.

As shown in Figure 1A, the effects of different ethanol concentrations (10, 20, 30, 40, 50, 60, 70, and 80%) on the extraction rate of polyphenols from *B. bicolor* were investigated at a fixed liquid-to-solid ratio of 35:1, an ultrasonic time of 40 min, and an ultrasonic temperature of 40°C. Figure 1A shows that the extraction rate of polyphenols first increases and then decreases with the ethanol concentration, reaching the maximum value at an ethanol concentration of 40%. It may be explained that the solubility of polyphenols is not high at lower ethanol concentrations; however, after the ethanol concentration increases to 40%, the dissolution ability of polyphenols may be hindered by certain competition effects, gradually reducing the solubility of polyphenols (21). Therefore, the optimal ethanol extraction concentration was determined to be 40%.

#### 3.1.2. Effect of the liquid-to-solid ratio on TPY

As shown in Figure 1B, the polyphenol extraction rate first increases and then decreases with the liquid-to-solid ratio, reaching the maximum value when the liquid-to-solid ratio is 35:1. The sample may not be completely immersed in the solution when the liquid-to-solid ratio is small. As the liquid-to-solid ratio increases, the contact area between the sample and the solution gradually rises until the sample is completely immersed, and the extraction rate of polyphenols dissolved in the solution gradually increases (26). After reaching a liquid-to-solid ratio of 35:1, the impurities dissolved in the solution gradually increase to inhibit the dissolution of polyphenols in the sample, so the extraction rate decreases (25). This indicates that when the solid-to-liquid ratio is too low, the excessive solvent absorbs more ultrasonic energy, reducing the energy absorbed by the mushroom material and, consequently, reducing the dissolution of the extract (27). Therefore, the optimal liquid–solid ratio was determined to be 35:1.

#### 3.1.3. Effect of the ultrasonication time on TPY

As shown in Figure 1C, when the ultrasonic time is 40 min, the polyphenol extraction rate reaches the maximum. It could be that the interaction of the sample and solution is insufficient at short ultrasonic times, so the extraction rate of polyphenols is not high; as the ultrasonic time increases, the extraction rate of polyphenols gradually increases. However, too long ultrasonication may damage the structure of polyphenols, reducing the extraction rate. Some polyphenols are sensitive to sonochemical reactions, such as oxidation, addition, degradation, and polymerization (27). Therefore, the optimal ultrasonic time is determined as 40 min.

#### 3.1.4. Effect of the temperature on TPY

The extraction yield of polyphenols exhibits a similar tendency to the ultrasonic temperature, Figure 1D. As the temperature increases above 40°C, the ethanol solvent evaporates, which may reduce the interaction between the sample and solution, suggesting that temperature is critical in the extraction rate of polyphenols (28). The temperature of the UAE process plays an important role in the extraction of bioactive compounds. These results might be related to the decrease in the surface tension and viscosity of the solvent, which induces an increase in vapor pressure. With the temperature increase, the sonochemical influence caused by the collapse of cavitation bubbles decreases, and polyphenols might degrade at higher temperatures. Therefore, keeping the temperature in an appropriate range is necessary during the extraction process of bioactive compounds. Therefore, the optimal ultrasonic temperature is determined to be 40°C.

### 3.2. Analysis of response surface methodology

A Box–Behnken experimental design was performed to determine the optimal extraction conditions using Design-Expert 11.1.1 software (29–32). The results are shown in Table 2. The quadratic regression model for the polyphenol extraction rate is obtained by fitting the regression to the data in Table 3 as follows:


$$\begin{array}{l} Y=-258.99+1.08X_{1}+7.34X_{2}+3.06X_{3}+3.03X_{4}\\ +0.0065X_{1}\times X_{2}-0.0013X_{1}\times X_{3}-0.0039X_{1}\times X_{4}\\ +0.011X_{2}\times X_{3}-0.021X_{2}\times X_{4}+0.016X_{3}\times X_{4}\\ -0.013X_{1}{}^{2}-0.103X_{2}{}^{2}-0.05X_{3}{}^{2}-0.034X_{4}{}^{2} \end{array}$$

**Table 2:** The experimental parameters and results of response surface analysis for extraction of polyphenol from *B. bicolor.*

Run	X1 (Ethnol concentration, %)	X2 (Liquid/Material ratio, mL/g)	X3 (Ultrasonic time, min)	X4 (Ultrasonic temperature, °C)	Polyphenols yield (mg/g)
1	40	35	40	40	13.3560 ± 0.9320
2	40	35	40	40	14.0602 ± 0.9760
3	40	35	40	40	13.5192 ± 0.8672
4	50	35	40	45	11.9800 ± 0.8561
5	40	30	40	35	10.1189 ± 0.8205
6	40	40	40	35	10.3484 ± 0.9821
7	40	40	45	40	10.1987 ± 0.8828
8	30	30	40	40	10.3937 ± 0.9702
9	40	40	35	40	9.10098 ± 0.9029
10	40	35	45	45	12.4165 ± 0.8222
11	40	30	35	40	10.1189 ± 0.9924
12	40	35	35	35	11.6744 ± 0.8320
13	40	35	40	40	13.4207 ± 0.9322
14	30	35	40	45	11.5870 ± 0.9823
15	40	35	45	35	11.3053 ± 0.9377
16	50	40	40	40	9.8993 ± 0.8865
17	40	40	40	45	8.9492 ± 0.9655
18	30	35	40	35	10.6702 ± 0.9388
19	50	35	40	35	11.8490 ± 0.9866
20	30	35	35	40	10.3646 ± 0.9658
21	40	30	40	45	10.8299 ± 0.8529
22	40	35	35	45	11.1068 ± 0.8699
23	50	30	40	40	10.0814 ± 0.7988
24	30	35	45	40	10.7180 ± 0.8643
25	40	30	45	40	10.0918 ± 0.8876
26	50	35	45	40	11.3687 ± 0.9302
27	40	35	40	40	14.0319 ± 0.9577
28	50	35	35	40	11.2814 ± 0.9688
29	30	40	40	40	8.9014 ± 0.9076

**Table 3:** ANOVA of the regression model.

Source	Sum of Square	df	Mean Square	*F* Value	*p* value	Significant
Model	57.66	14	4.12	41.47	<0.0001	significant
X1-(Ethanol concentration)	1.22	1	1.22	12.28	0.0035	
X2-(Solvent/material ratio)	1.50	1	1.50	15.06	0.0017	
X3-(Ultrasonic time)	0.5011	1	0.5011	5.05	0.0414	
X4-(Ultrasonic temperature)	0.0680	1	0.0680	0.6847	0.4218	
X1X2	0.4291	1	0.4291	4.32	0.0565	
X1X3	0.0177	1	0.0177	0.1782	0.6793	
X1X4	0.1544	1	0.1544	1.55	0.2329	
X2X3	0.3163	1	0.3163	3.18	0.0960	
X2X4	1.11	1	1.11	11.21	0.0048	
X3X4	0.7046	1	0.7046	7.09	0.0185	
X1^2^	11.68	1	11.68	117.58	<0.0001	
X2^2^	43.83	1	43.83	441.36	<0.0001	
X3^2^	10.30	1	10.30	103.74	<0.0001	
X4^2^	4.96	1	4.96	49.91	<0.0001	
Residual	1.39	14	0.0993			
Lack of fit	0.9239	10	0.0924	0.7923	0.6531	not significant
Pure error	0.4664	4	0.1166			
Cor total	59.05	28				
*R* ^2^	0.9765					
*R*^2^_adj_	0.9529					

Where Y denotes the corresponding value indicating the polyphenol extraction rate (mg/g). The model validity was confirmed using ANOVA and presented in Table 3. The *F*-value is 41.47 (*p* < 0.0001), indicating that the model is highly significant. The determination coefficient *R*^2^ is 0.9765, indicating a high correlation between the experimental and predicted values (33–37). The adjusted determination coefficient *R*^2^_adj_ is 0.9529, implying that the model can explain 95.29% of the variation in response values. Therefore, the model can be used to predict and analyze the polyphenol extraction from *B. bicolor*. All correlation coefficients indicate that the responses are in very good agreement with the predicted extraction yields.

**Figure 2:**
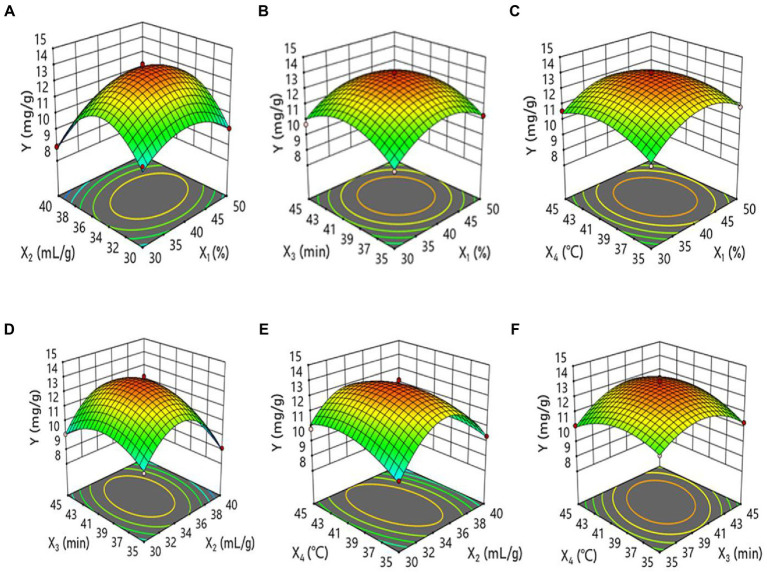
Response surface plots of the effects of **(A)**; ethanol concentration and extraction time (min) **(B)**; and solvent/material ratio and extraction time.

The three-dimensional response surfaces illustrate the relationship between independent and dependent variables. Figure 2 shows the graphs representing the effects of different ethanol concentrations, liquid-to-solid ratios, ultrasonic times, and ultrasonic temperatures. The value of *p* shows that the interaction is highly significant for X_2_X_4_ (liquid-to-solid ratio and ultrasonic temperature factors) and significant for X_3_X_4_ (ultrasonic time and ultrasonic temperature). In other words, there is a significant interaction between the liquid-to-solid ratio and ultrasonic temperature factors. The surface plot between ultrasonic time and ultrasonic temperature factors is also steep, indicating that the two variables exhibit significant interaction. Furthermore, the order of the interaction is X_2_X_4_ > X_3_X_4_ > X_1_X_2_ > X_2_X_3_ > X_1_X_4_ > X_1_X_3_.

### 3.3. Comparison of ultrasound-assisted extraction with soxhlet and maceration extraction methods

To estimate and validate the efficiency of ultrasound on polyphenol extraction from *B. bicolo*r, we compared it with conventional extraction methods (soxhlet and maceration). As seen from Table 4, the extraction rate of polyphenols by the ultrasound-assisted method is significantly higher than for the other two traditional methods, i.e., the extraction rate is 1.7 times higher than that of soxhlet extraction and 2.1 times higher than that of maceration extraction. Thus, the UAE requires less time to achieve better efficiency. The UAE method can be a potential alternative to conventional extraction methods since it improves the yield and quality of the extract.

**Table 4:** The comparison of UAE with maceration and soxhlet extraction.

Extracting method	Ethanol concentration	Temperature	Time	Phenolic yield/(mg/g)
UAE	42%	40°C	41 min	13.69 ± 0.17
Soxhlet extraction	42%	95°C	4 h	7.98 ± 0.23
Maceration extraction	42%	25°C	24 h	6.65 ± 0.34

### 3.4. Qualitative and quantitative determination of polyphenolic compounds

**Figure 3:**
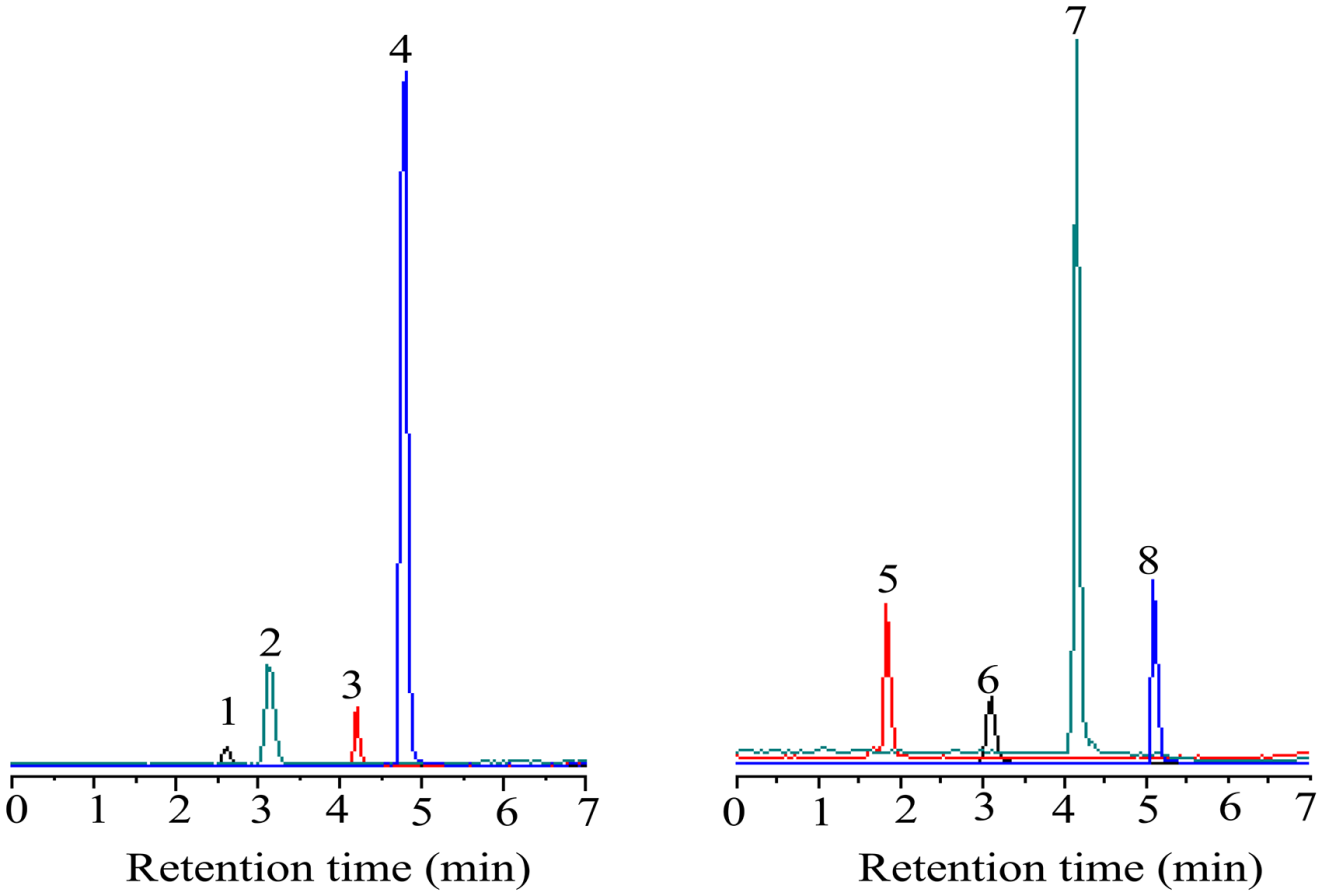
The diagram of eight standard phenolic compounds (1- protocatechuic acid; 2-Catechin 3-Syringic acid; 4-Rosmarinic acid; 5-Gallic acid; 6-Chlorogenic acid; 7-Caffeic acid; and 8-Quercetin).

**Figure 4:**
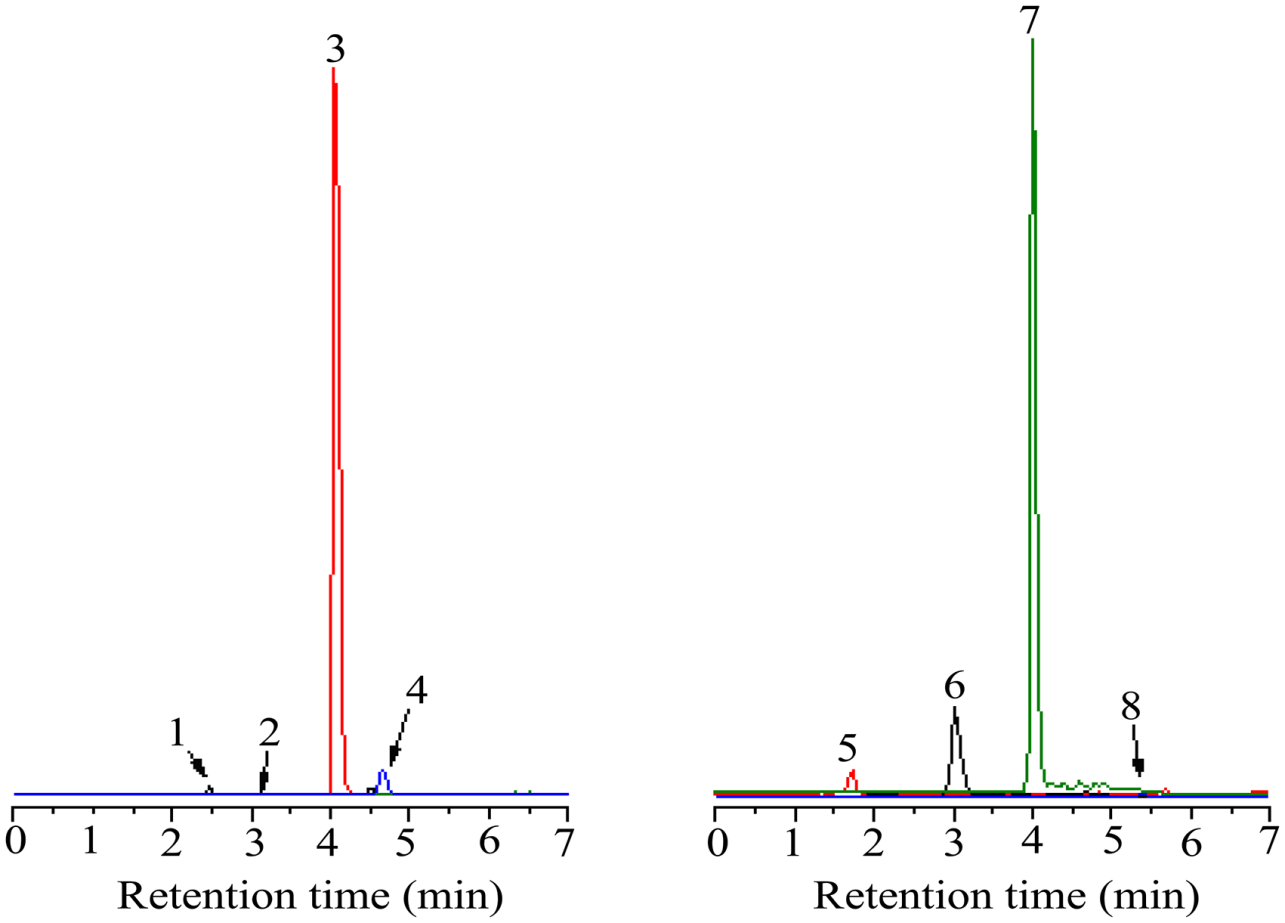
The diagram of eight kinds of phenolic compounds from *Boletus bicolor.*

**Figure 5:**
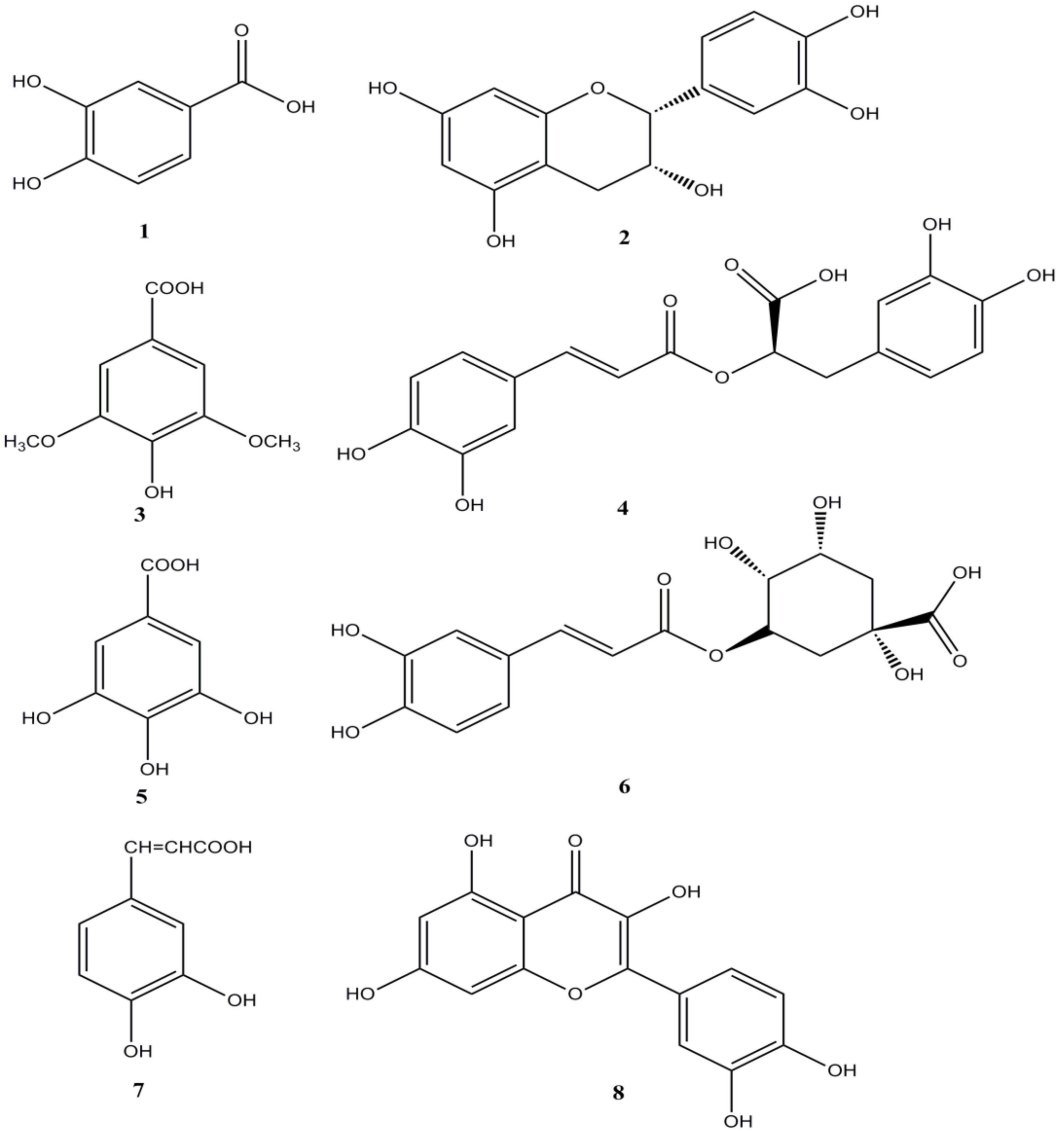
The eight compound structures of from *Boletus bicolor.*

In this study, eight polyphenols were identified using UPLC-MS. As shown in Figures 3, 4 and Tables 5–7, the eight polyphenols identified are protocatechuic acid, catechin, syringic acid, rosmarinic acid, chlorogenic acid, gallic acid, caffeic acid, and quercetin. In Table 7, the content of syringic acid is the highest, followed by chlorogenic acid, caffeic acid, protocatechuic acid, gallic acid, rosmarinic acid, catechin, and quercetin in the polyphenolic extract of *B. bicolor*, which may be the main substances with antioxidant activity in the polyphenolic extract of this mushroom. In other words, the strong antioxidant activity of the extract may be related to these polyphenolic ingredients that act as free radical scavengers (Figure 5).

**Table 5:** The parent ion and daughter ions of the eight moleculars.

Analyte	Molecular formula	Parent ion (m/z)	Daughter ions (m/z)	Declustering potential (V)	Collsision energy (V)
Gallic acid	C_7_H_6_O_5_	168.9	124.8	−60	−19
Caffeic acid	C_9_H_8_O_4_	179.0	135.0	−42	−23
Syringic acid	C_9_H_10_O_5_	197.2	121.0	−50	−24
Chlorogenic	C_16_H_18_O_9_	353.1	191.2	−64	−33
Catechin	C_15_H_14_O_6_	289.0	245.0	−77	−23
Quercetin	C_15_H_10_O_12_	301.0	151.0	−86	−30
Rosmarinic acid	C_18_H_16_O_8_	359.0	161.0	−57	−20
Protocatechuic acid	C_7_H_6_O_4_	153.0	90.6	−56	−29

**Table 6:** The linearity and range, detection limits, and quantification limits of the standard curves for the eight phenolic compounds from *B. bicolor.*

Phenolic compounds	Linear range/(ng/mL)	Linear equations	*r* ^2^	Detection limits/(ng•mL^-1^)	Quantitative limits /(ng•mL^-1^)
Gallic acid	10.3 ~ 206	y = 3531.64281x-26890.46117	0.9995	4.60	15.3
Caffeic acid	1.02 ~ 204	y = 12805.50583x + 4345.93685	0.9999	6.59	22.0
Quercetin	1.03 ~ 103	y = 3510.38441x-1540.83935	0.9984	1.21	4.03
Rosmarinic acid	1.04 ~ 208	y = 4060.92813x-9408.46058	0.9993	0.766	2.55
Protocatechuic acid	2.06 ~ 515	y = 126.74091x + 126.76760	0.9997	10.5	35.0
Chlorogenic acid	1.06 ~ 530	y = 1772.47142x + 3324.15831	0.9995	0.782	2.61
Syringic acid	1.04 ~ 520	y = 234.19641x + 537.63508	0.9999	5.02	16.7
Catechin	1.00 ~ 200	y = 846.12342x-947.76446	0.9992	13.5	45.0

**Table 7:** Polyphenols content in *B. bicolor* (μg·g ^−1^).

Kinds of polyphenols	Gallic acid	Caffeic acid	Quercetin	Rosmarinic acid
Polyphenol content	7.20	42.07	0.59	3.13
Kinds of polyphenols	Protocatechuic acid	Chlorogenic acid	Syringic acid	Catechin
Polyphenol content	24.76	55.35	1045.79	1.29

### 3.5. DFT analysis of polyphenolic antioxidant activities

**Figure 6:**
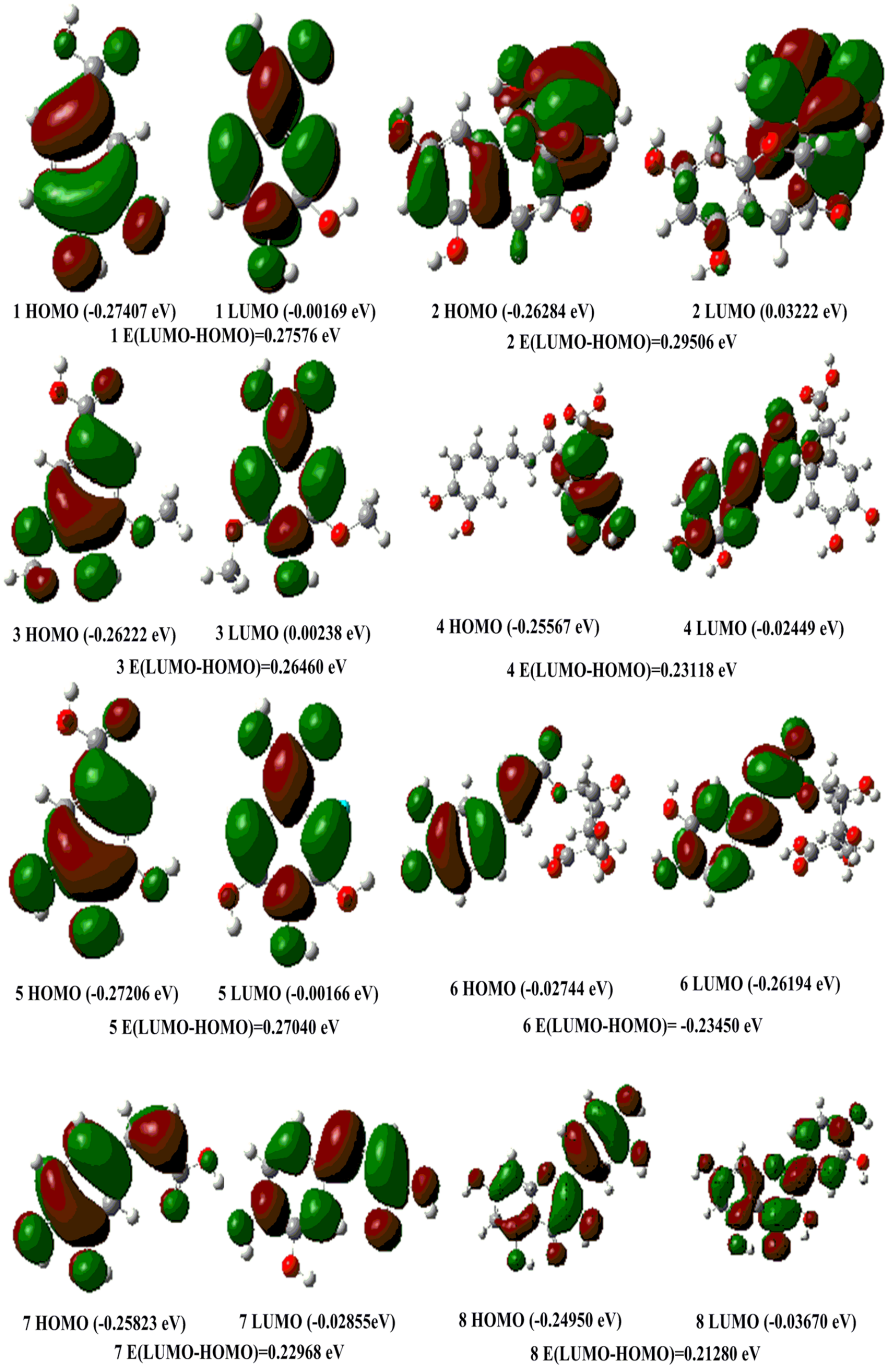
The HOMO and LUMO orbits of eight compound structures in *Boletus bicolor.*

The energies and distribution of molecular frontier orbitals could provide useful information for evaluating antioxidant activities. The molecular orbital theory in quantum chemistry considers that the distribution of the highest occupied molecular orbital (HOMO) and the lowest unoccupied molecular orbital (LUMO) can visualize the main action sites of antioxidants in scavenging-free activity (37). The energy level difference of frontline molecular orbitals is an important theoretical parameter to characterize molecular activity. The higher the HOMO energy, the stronger the electron-donating ability (37). The smaller the LUMO energy, the stronger the electron-withdrawing ability. The energy and distribution of frontier orbitals are useful descriptors in evaluating the antioxidant properties of phenolic compounds. The energy gap can accurately characterize the order of antioxidant activities of molecules. The smaller the HOMO-LUMO energy gap is, the more readily the electron promotion occurs. A molecule with a higher HOMO energy exhibits a stronger electron-donating ability, indicating high antioxidant activity. Figure 6 shows the HOMO and LUMO energies distribution for the eight polyphenolic compounds. We can see that the order of the HOMO-LUMO energy gap is chlorogenic acid < quercetin < caffeic acid < rosmarinic acid < syringic acid < gallic acid < protocatechuic acid < catechin. On the other hand, the orbital energy difference (ΔE) for the studied polyphenolic compounds is also given in Figure 6. Chlorogenic acid exhibits the highest HOMO energy (−0.02744 eV) and the lowest energy difference (−0.23450 eV) among the studied compounds, suggesting that the compound might be the strongest antioxidant molecule. The highest energy difference is found for catechin (ΔE = 0.29506 eV). Indicating that catechin might be the weakest electron-donating ability, respectively.

### 3.6. Polyphenolic compounds inhibitory effect on intracellular ROS generation

**Figure 7:**
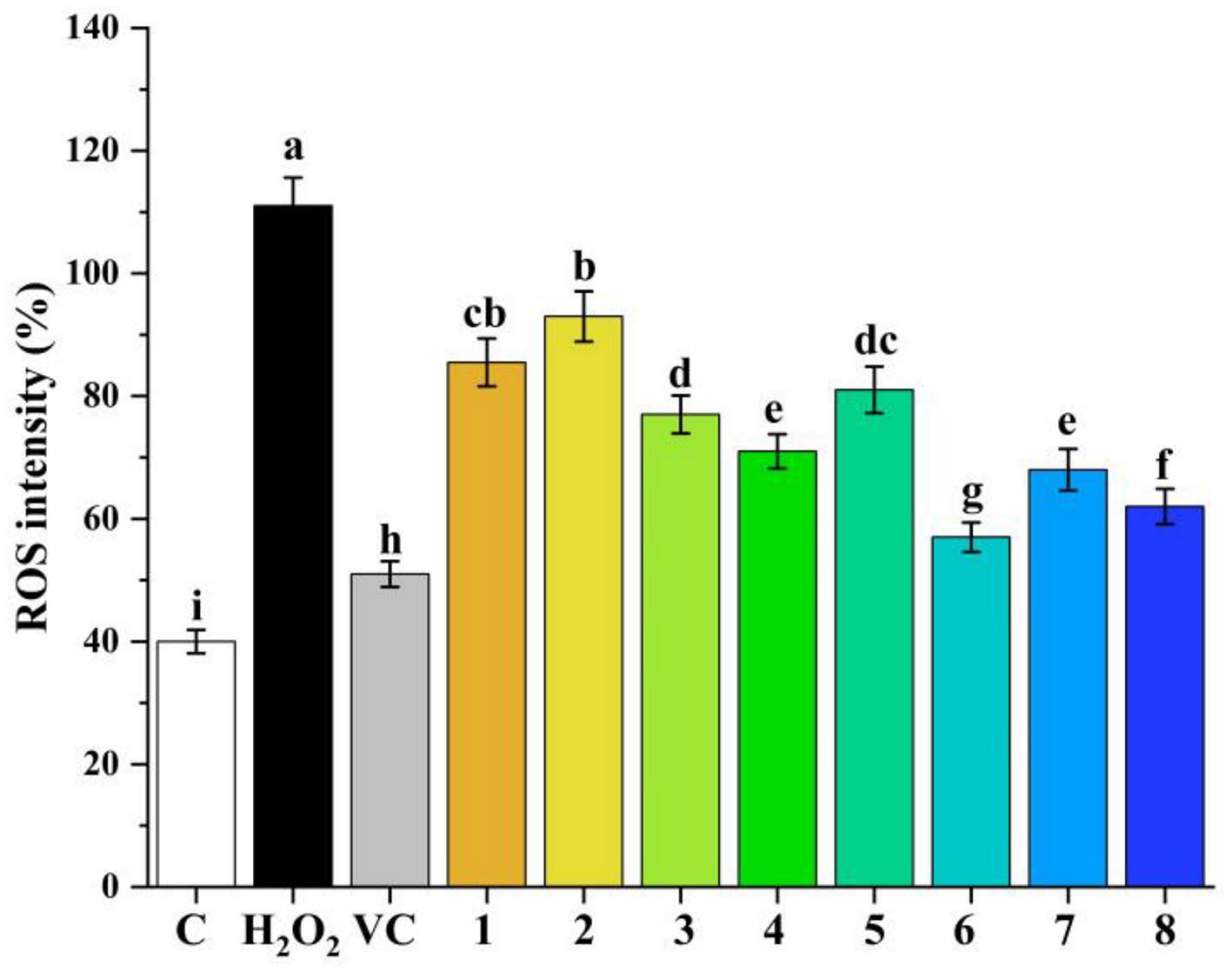
Cellular ROS inhibitory effects of phenolic compounds (1–8) in H_2_O_2_-induced HepG2 cells (1-protocatechuic acid; 2-Catechin 3-Syringic acid; 4-Rosmarinic acid; 5-Gallic acid; 6-Chlorogenic acid; 7-Caffeic acid; and 8-Quercetin). **(A)** DPPH scavenging capacity. **(B)** ABTS scavenging capacity. **(C)** OH scavenging activity. **(D)** NO_2_^−^ scavenging capacity.

To further explore the antioxidant capacity of eight polyphenolic compounds, intracellular ROS production was measured in H_2_O_2_-induced HepG2 cells. The H_2_O_2_-induced HepG2 cell model has been reported to be a classical model for assessing the antioxidant capacity of compounds (38) ROS play an important role in normal physiological functions and human diseases; the accumulation of high levels of ROS *in vivo* leads to protein and lipid oxidation, cellular damage, cellular aging, and even apoptosis (39). As shown in Figure 7, Compared with the control group, H_2_O_2_ administration significantly induced intracellular ROS production (*p* < 0.05). However, the intervention of eight polyphenolic compounds greatly reduced the H_2_O_2_-induced intracellular ROS production in HepG2 cells compared to the H_2_O_2_ group (*p* < 0.05). Moreover, the antioxidant capacity of the eight polyphenolic compounds were ranked as chlorogenic acid > quercetin > caffeic acid > rosmarinic acid > gallic acid > protocatechuic acid > catechin. The above results once again proved DFT analysis of the eight polyphenolic compounds.

### 3.7. Evaluation of antioxidant activity

It was reported that the antioxidant activity of phenolic compounds in biological systems is the most important component of their bioactivity, where they act as free radical inhibitors (40–42). Eight mushrooms, including *Agaricus bisporus*, *Boletus edulis*, *Calocybe gambosa*, *Cantharellus cibarius*, *Craterellus cornucopioide*s, *Hygrophorus marzuolus*, *Lactarius deliciosus*, and *Pleurotus ostreatus* were evaluated, and the phenolic compounds have shown a significant inhibition effect against lipid oxidation (40). Yahia et al. analyzed the phenolic compounds of 17 species of wild mushrooms and detected their antioxidant activities by FRAP and DPPH assays, suggesting an effective nutritional and health value of different mushroom species (41). Hu et al. investigated the neuroprotection of six components from *Flammulina velutipes*, which were mainly phenolic compounds, on H_2_O_2_^−^induced oxidative damage in PC12 cells and demonstrated that most of the components exhibit neuroprotective effects along with their antioxidant activities (42). To evaluate the antioxidant activities of *B. bicolor*, we performed five antioxidant activities experiments as follows:

#### 3.7.1. Assay of DPPH scavenging activity

**Figure 8:**
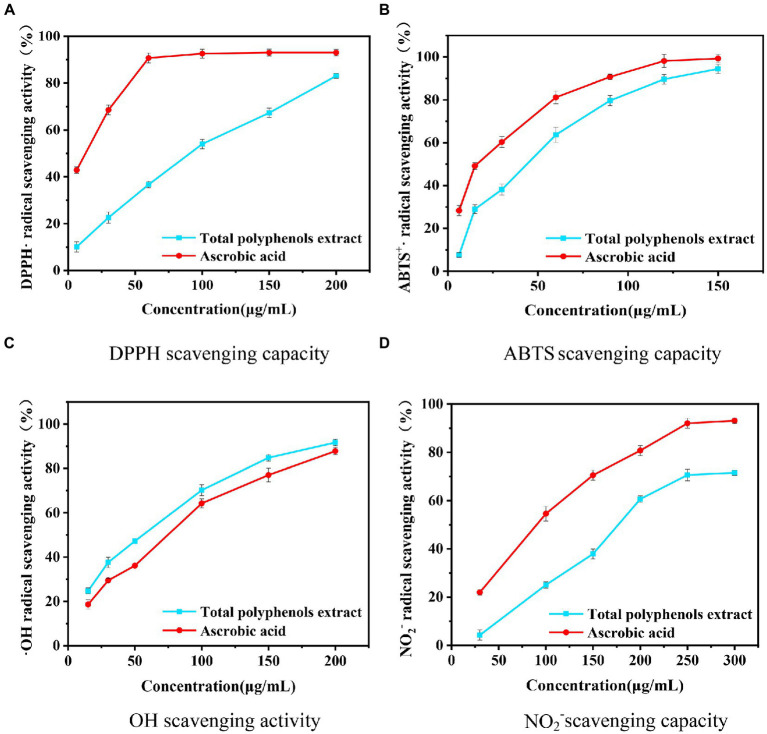
Evaluation of the effect of five methods on different concentrations of *Boletus bicolor* polyphenols.

As seen from Figure 8, the DPPH scavenging activities of the sample and ascorbic acid increase with the mass concentration, and the scavenging ability of the sample is slightly lower than that of ascorbic acid. The sample and ascorbic acid clearances reach 83.07 and 93.10%, respectively. The IC_50_ of the sample is 98.17 μg/mL, and the IC_50_ of ascorbic acid is 19.50 μg/mL. The DPPH scavenging rate of polyphenols may be due to different types of phenolic acids contained in the samples (43–45).

#### 3.7.2. Assay of ABTS scavenging activity

The results of ABTS scavenging ability are shown in Figure 8. The scavenging ability of the sample is weaker compared to ascorbic acid (40–42, 46–48). At a concentration of 150 μg/mL, the *B. bicolor* extract exhibits 94.45% inhibition, while it is 99.53% for ascorbic acid. The IC_50_ value of the *B. bicolor* extract is 49.80 μg/mL, while it is 21.93 μg/mL for ascorbic acid. Compared to the DPPH scavenging ability, it is improved twice, indicating that polyphenol substances in the *B. bicolor* extract are great ABTS scavengers.

#### 3.7.3. OH scavenging activity

Salicylic acid can capture hydrogen peroxide (H_2_O_2_), and Fe^2+^ can generate an OH radical reaction to produce colored substances with a characteristic absorption at a wavelength of 510 nm. Phenolic antioxidants exhibit the effect of scavenging OH radicals, reducing the generation of colored substances and lowering the absorbance value, so the antioxidant capacity can be estimated based on the absorbance to determine the level of antioxidant activity (49). Figure 8 showed that the *B. bicolor* extract exhibits better OH radical scavenging activity than ascorbic acid at different concentrations. In particular, at a concentration of 350 μg/mL, the *B. bicolor* extract exhibits the maximum scavenging of 93.76%, comparable to the ascorbic acid scavenging of 88.83%. The IC_50_ value of the *B. bicolor* extract is 44.23 μg/mL, and for the ascorbic acid, it is 60.79 μg/mL. The ability of the *B. bicolor* extract to scavenge OH radicals is 1.37 times higher than that of ascorbic acid, indicating the great performance of the *B. bicolor* extract polyphenols on scavenging OH radicals.

#### 3.7.4. NO_2_^−^ scavenging activity

Nitrosamines are the most potent carcinogenic chemicals in humans. Nitrites are the source of nitrosamines, so removing nitrites may effectively prevent cancer in humans (50–52). Nitrites react with p-aminobenzenesulfonic acid and then couple with naphthylenediamine hydrochloride to form a red complex. When antioxidants are present in the system, they remove nitrites, making the system less colored. Figure 8 shows that the ability of the sample and ascorbic acid to scavenge NO_2_^−^ increases with the mass concentration, and the scavenging ability of the sample is weaker than that of ascorbic acid. Especially, the *B. bicolor* extract exhibits the maximum scavenging of 76.2%, comparable to the NO_2_^−^ scavenging activity of ascorbic acid. The IC_50_ value of ascorbic acid is 94.77 μg/mL, and that of the *B. bicolor* extract is 174.46 μg/mL, indicating that the polyphenols in the *B. bicolor* extract exhibit a certain scavenging effect on NO_2_^−^.

#### 3.7.5. Ferric reducing antioxidant power

**Figure 9:**
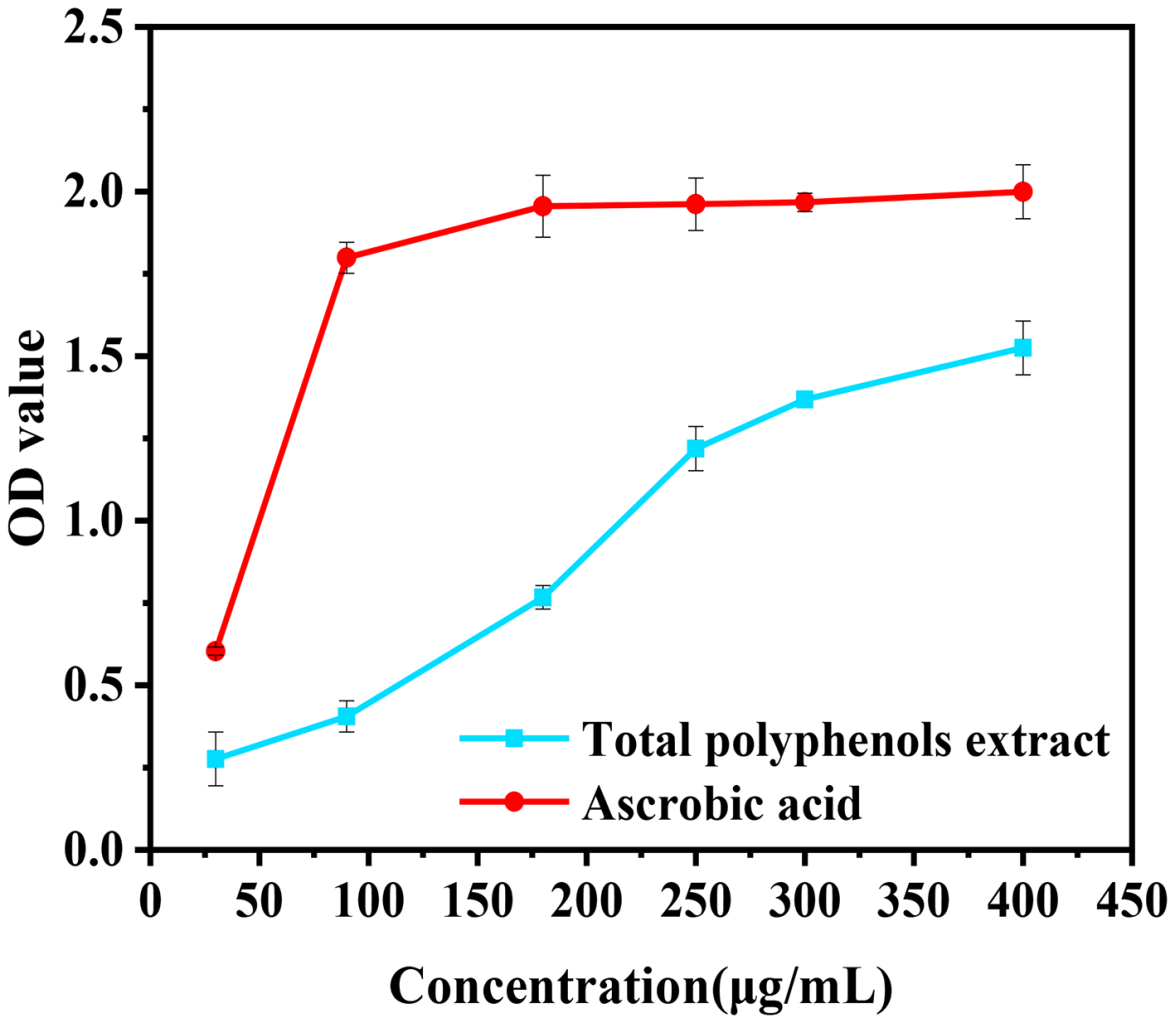
Reduction ability of polyphenols from *Boletus bicolor* to iron ion.

The reducing ability is used as an indicator to convert Fe^3+^ to Fe^2+^. The presence of antioxidants in the system might cause the conversion of Fe^3+^ to Fe^2+^ in potassium ferricyanide to form a blue-green complex, and the stronger the reducing ability of the antioxidants, the greater their absorbance values (51, 52). From Figure 9 can be seen that the ability of the sample and ascorbic acid to reduce ferrous ions increases with the mass concentration. The sample behavior becomes weaker relative to ascorbic acid when the mass concentration reaches 180 μg/mL. When the mass concentration reaches 250 μg/mL, the absorbance value of the sample is 1.218. At a mass concentration of 400 μg/mL, the absorbance values of ascorbic acid and the sample are 1.525 and 1.999, respectively. Thus, the EC_50_ of the sample and ascorbic acid are 123 and 48 μg/mL, respectively, and the reducing power of the sample is 0.39 times that of ascorbic acid.

## 4. Conclusion

This paper optimized the extraction process of polyphenols from *B. bicolor* using the RSM method. Based on the response surface analysis, the optimum extraction conditions were determined: an ethanol concentration of 42%, a solvent-to-solid ratio of 34:1 mL/g, a temperature of 40°C, and an ultrasonic time of 41 min. The polyphenol extraction rate was 13.69 ± 0.13 mg/g with a relative deviation of 0.2%. The advantages of the method in terms of time-saving and high efficiency were compared with the traditional soxhlet extraction. Besides, the antioxidants activities of mushroom extracts exhibited good 2, 2-dipheny-1-picrylhydrazyl radical (DPPH·), 2, 2-azino-bis (3-ethylbenzothiaziline-6-sulfonic acid) radical (ABTS^+^·), hydroxyl radical (OḤ), and nitric radical (NO_2_^−^) scavenging activities, as well as the ferric reducing antioxidant power. Eight main phenolic compounds found in the extract of *B. bicolor* were identified and quantified by the UPLC-MS method: protocatechuic acid, catechin, syringic acid, rosmarinic acid, gallic acid, chlorogenic acid, caffeic acid, and quercetin. Additionally, the DFT calculations showed that chlorogenic acid exhibited the highest HOMO energy and the lowest energy difference among the studied compounds, while the lowest HOMO energy and the highest energy difference were found for quercetin. The intervention of eight polyphenolic compounds greatly reduced the H_2_O_2_-induced intracellular ROS production in HepG2 cells as compared to the H_2_O_2_ group, and showed strong cell protective effects. The antioxidant capacity of the eight polyphenolic compounds proved the DFT analysis. The results provide theoretical guidance for developing natural antioxidants based on *B. bicolor* resources. This study might be useful for future developments and pharmaceutical applications of polyphenols in *B. bicolor* mushrooms.

## Data availability statement

The original contributions presented in the study are included in the article/supplementary material, further inquiries can be directed to the corresponding authors.

## Author contributions

S-LS and X-SZ conceived and designed the experiment and contributed to valuable discussion and revising manuscript. D-BH and RX performed the experiments. X-CZ analyzed the data. D-BH wrote the paper. All authors contributed to the article and approved the submitted version.

## Funding

This work was supported by the National Natural Science Foundation of China (No. 31960480) and Regional Universities Joint Special Project Surface Project (202101BA070001-193).

## Conflict of interest

The authors declare that the research was conducted in the absence of any commercial or financial relationships that could be construed as a potential conflict of interest.

## Publisher’s note

All claims expressed in this article are solely those of the authors and do not necessarily represent those of their affiliated organizations, or those of the publisher, the editors and the reviewers. Any product that may be evaluated in this article, or claim that may be made by its manufacturer, is not guaranteed or endorsed by the publisher.
